# Supplementary material for: Pooled library screening with multiplexed Cpf1 library
Source: Nat Commun. 2019 Jul 17;10:3144. doi: 10.1038/s41467-019-10963-x (PMC6637147; doi:10.1038/s41467-019-10963-x)
Supplement: Supplementary file 1 — Supplementary Information [file 41467_2019_10963_MOESM1_ESM.pdf]

Supplementary Information

Pooled library screening with multiplexed Cpf1 library

Liu et al

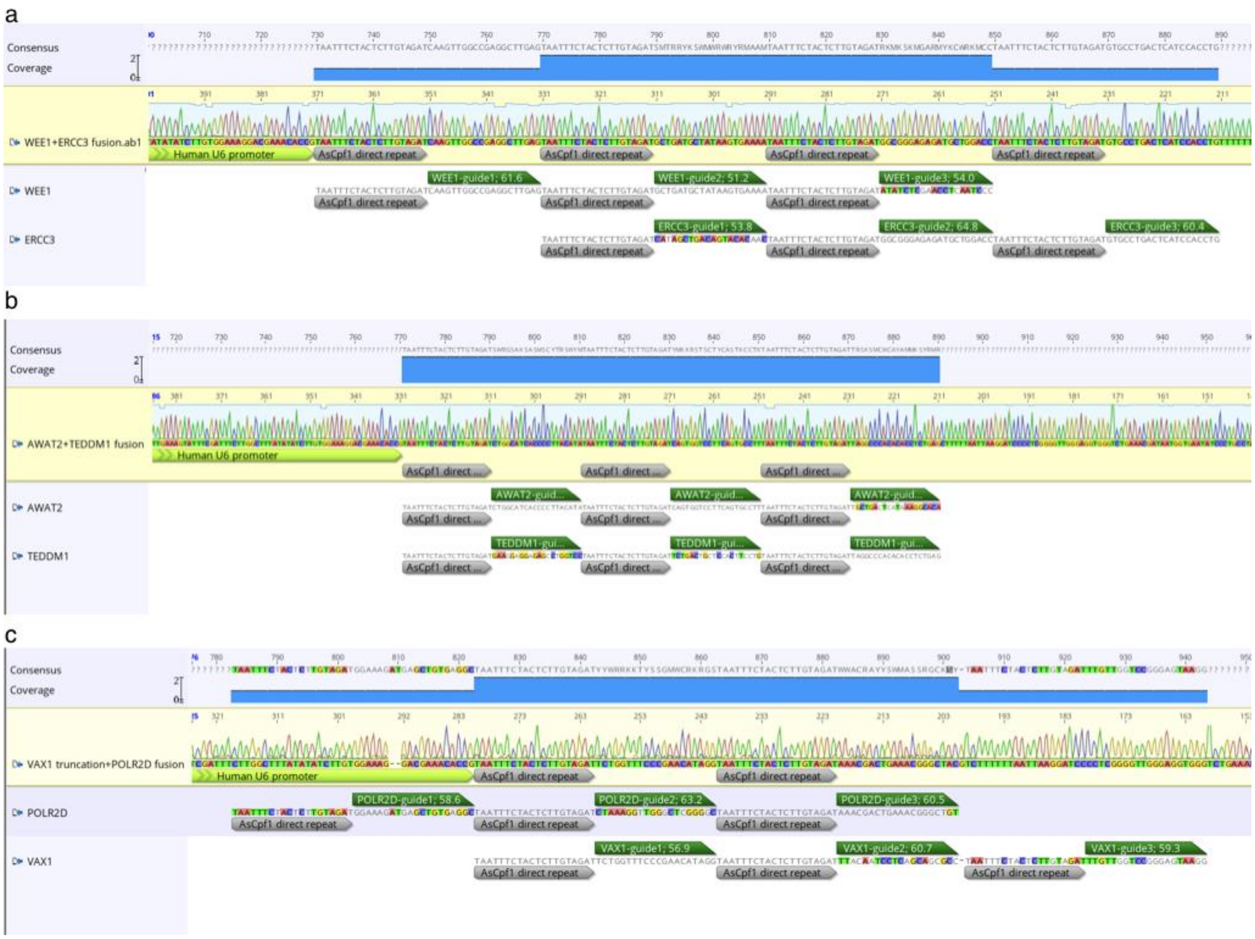

Supplementary Figure 1 Examples of amplicon rearrangement caused by Gibson assembly.

Sanger sequencing results of randomly picked colonies showing rearranged molecule generated from 2 different 3-guide array amplicon, resulting 4-guide hybrid **(a),** 3-guide hybrid **(b),** and truncated 2-guide hybrid**(c).**

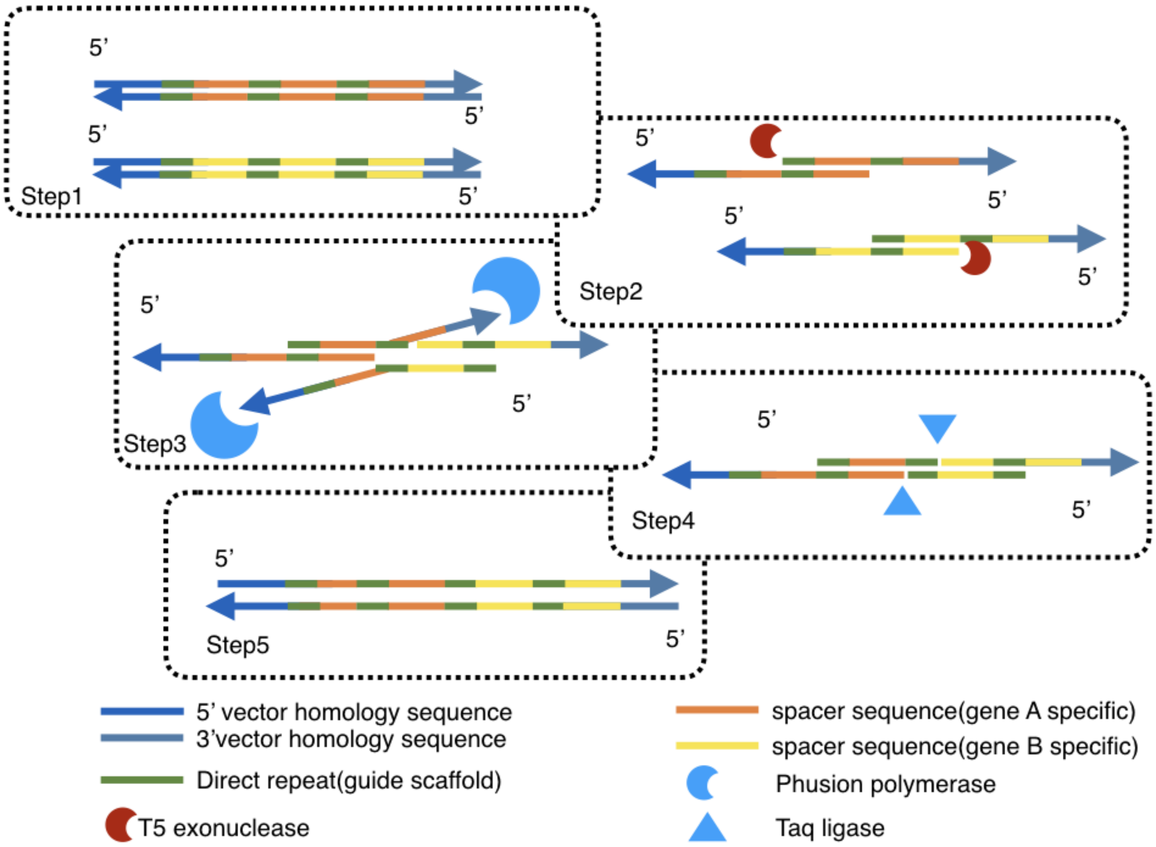

Supplementary Figure 2 Proposed amplicon rearrangement caused by Gibson assembly.

After PCR amplicon of oligo duplexes are added into Gibson assembly reaction (step 1), T5 exonuclease starts to erode the duplex from both strands’ 5’ terminals, exposing the direct repeat sequence of AsCpf1 guides as single strand ready for hybridization (step 2). Different guide arrays hybrid together because of homology in guides’ direct repeats, leaving a long imperfect tail. Phusion polymerase binds to the tails and use its 3’->5’ exonuclease activity to degrade the tails until it meets the perfect hybridization regions consisting of guide direct repeats (step3). Taq polymerase seals nicks between the hybrid chains (step 4) and the recombined molecule is generated and cloned into vector (step 5).

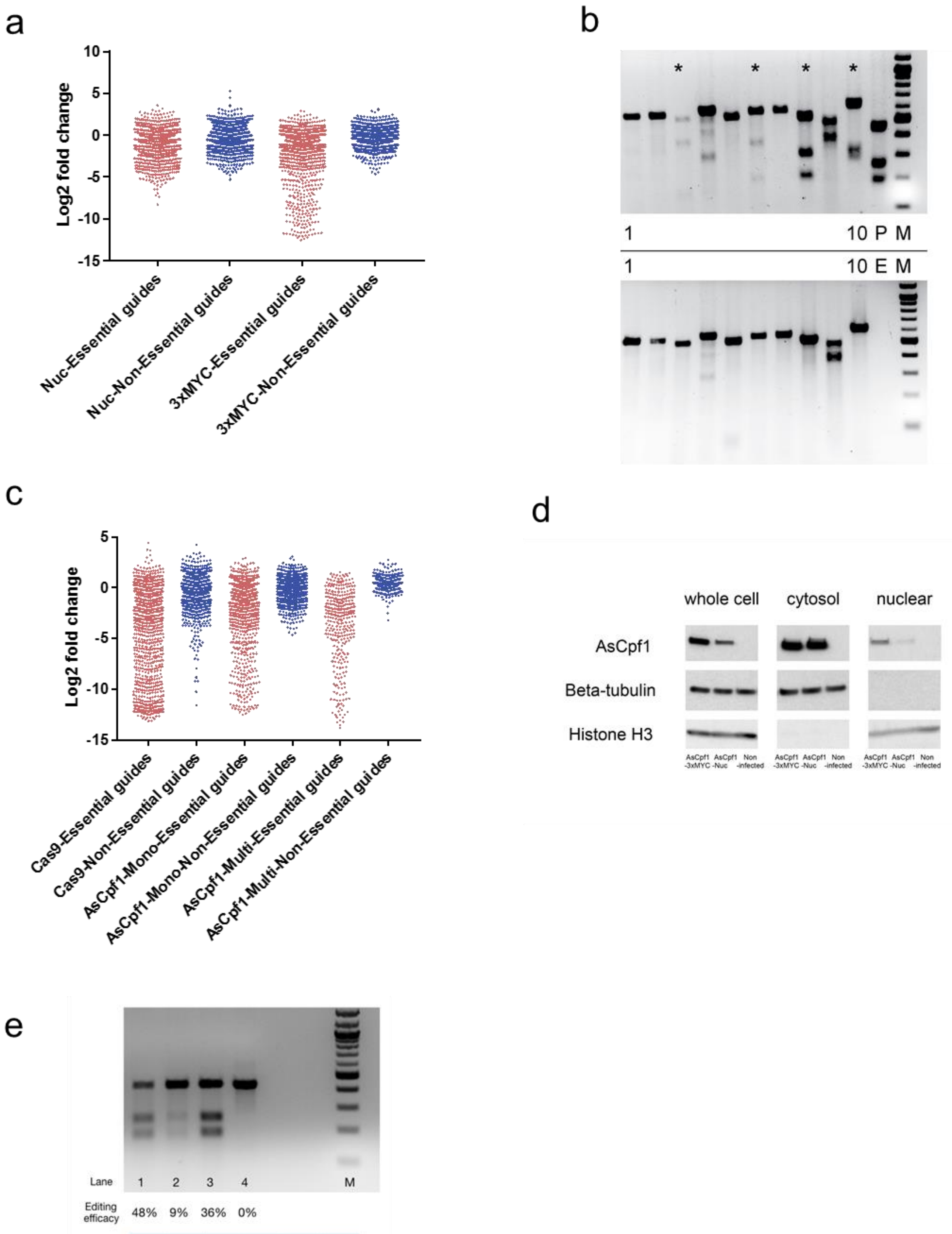

Lane	1	2	3	4	M
AsCpf1 variant	AsCpf1-3 xMYC	AsCpf1-Nuc	AsCpf1-SV40Nuc	No AsCpf1	Marker
N-NLS	3xMYC	-	2xSV40	N.A	N.A
C-NLS	-	1xNuc	1xNuc	N.A	N.A

Supplementary Figure 3 AsCpf1 optimization for functional genomics

a. Depletion of essential and non-essential gene targeting guide in AsCpf1-nuc variant and AsCpf1-3xMYC based screen. Pink: the mean value of log2 transformed fold change of essential gene targeting guides at the endpoint, n=1026. Purple: the mean value of log2 transformed fold change of non-essential gene targeting guides at the endpoint, n=1035. **b.** T7E1 assay of 10 different loci transfected with AsCpf1-nuc and corresponding guides. Active guides are indicated by "*". Upper panel: cells transfected with AsCpf1-nuc and guide. Lower panel: cells transfected with guides only. Lane1 to lane 10: T7E1 assay products targeting loci (from left to right) RESP18, NEUROD6, NAMPT, PRAMEF2, SLC22A9, COX7B2, TFAP2D, GUCY2F, ADAM30, and FNDC9. P: T7E1 assay product of DNMT1 edited allele as a positive control for T7 endonuclease activity. E: Empty lane. M: NEB 100bp DNA ladder. **c.** Depletion of essential and non-essential gene targeting guide in AsCpf1-3xMYC and SpCas9 based screen. Pink dot: the mean value of log2 transformed fold change of essential gene targeting guides at the endpoint, n=1026 for SpCas9 and AsCpf1 based mono-cistronic library, n=342 for AsCpf1 based multiplexed library. Purple dot: the mean value of log2 transformed fold change of non-essential gene targeting guides at the endpoint, n=1035 for SpCas9 and AsCpf1 based mono-cistronic library, n=345 for AsCpf1 based multiplexed library. **d.** Western blot of AsCpf1 variant. K-562 cells were infected with AsCpf1-Nuc (the commonly used variant) and AsCpf1-3xMYC (Kozak sequence and nucleus localization signal optimized variant) virus at the same multiplicity of infection **e.** T7E1 assay results of AsCpf1 variants on locus DNMT1. HEK293-T cells were transfected with different AsCpf1 variants and guide targeting DNMT1 locus (lane 1-3) in complexed with lipofectamine 3000. 3xMYC (3x tandem MYC NLS), 2x SV40 (2x tandem SV40 NLS), 1xNuc(1x Nucleoplasmin NLS), N.A.(Not applicable). Lane 4: negative control with guide transfection only. Lane M: NEB 100bp DNA ladder. Source data are provided as a Source Data file.

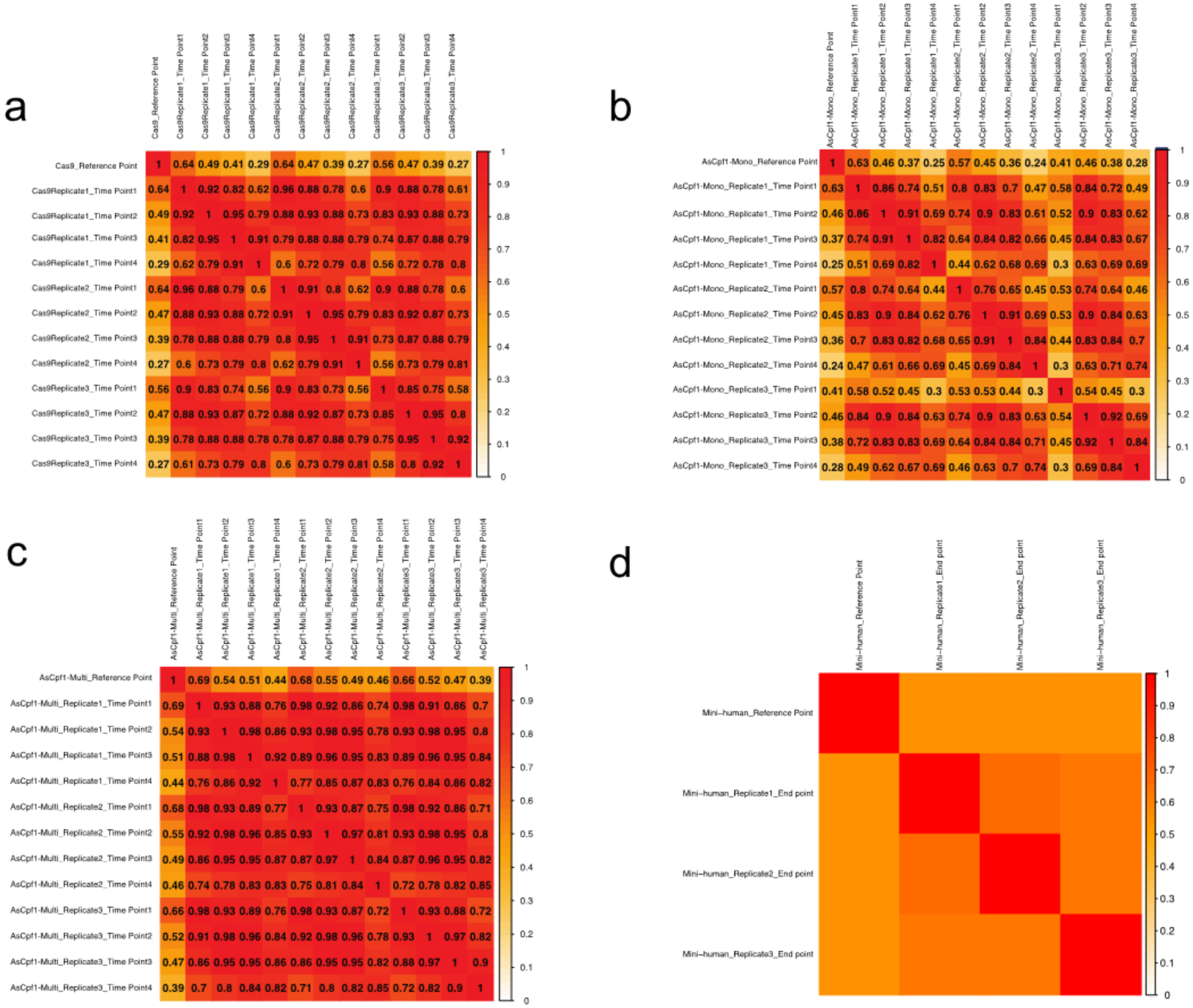

Supplementary Figure 4 Correlation of replicates in the pooled screenings

Pearson correlation coefficient matrices of all samples for SpCas9 based mono-cistronic benchmark library screening **(a)**, AsCpf1 based mono-cistronic benchmark library screening **(b).** AsCpf1 based multiplexed benchmark library screening **(c).** Mini-human genome-wide screening **(d)**. Raw reads counts were used for the analysis. Source data are provided as a Source Data file.

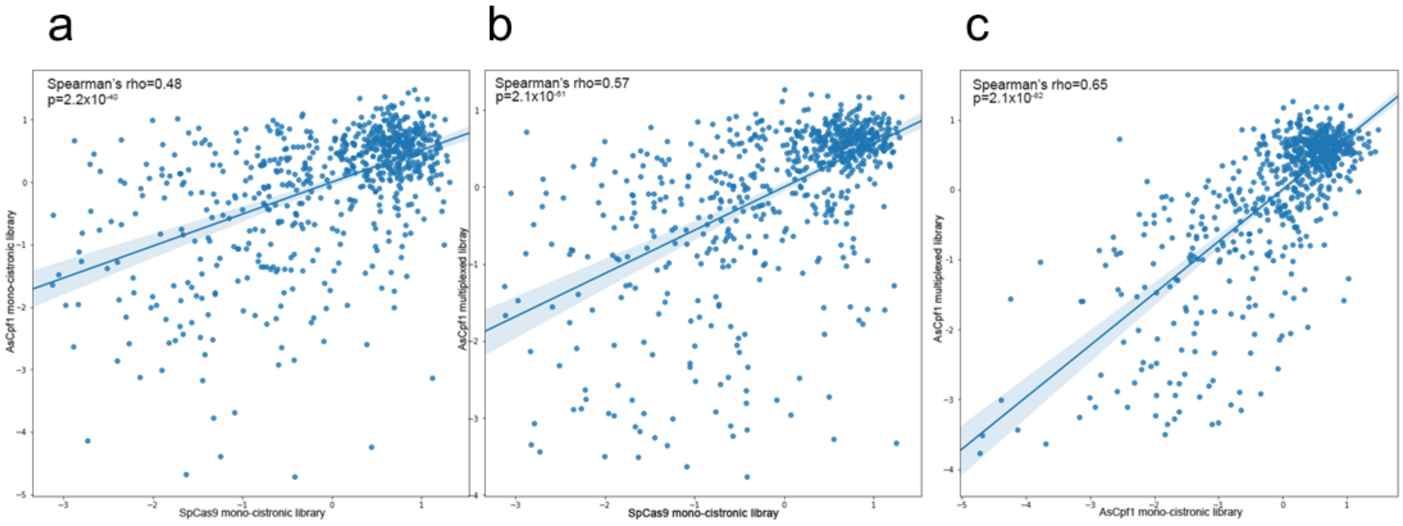

Supplementary Figure 5 Correlation of hit's ranks between different CRISPR libraries

Rank's correlations between benchmark library AsCpf1-Mono vs Cas9 **(a)**, AsCpf1-Multi vs Cas9 **(b)**, AsCpf1-Multi vs AsCpf1-Mono **(c).** Z scores of core-essential genes from each library were used. Line of best fit is plotted and the shaded area is presented as SD. Source data are provided as a Source Data file.

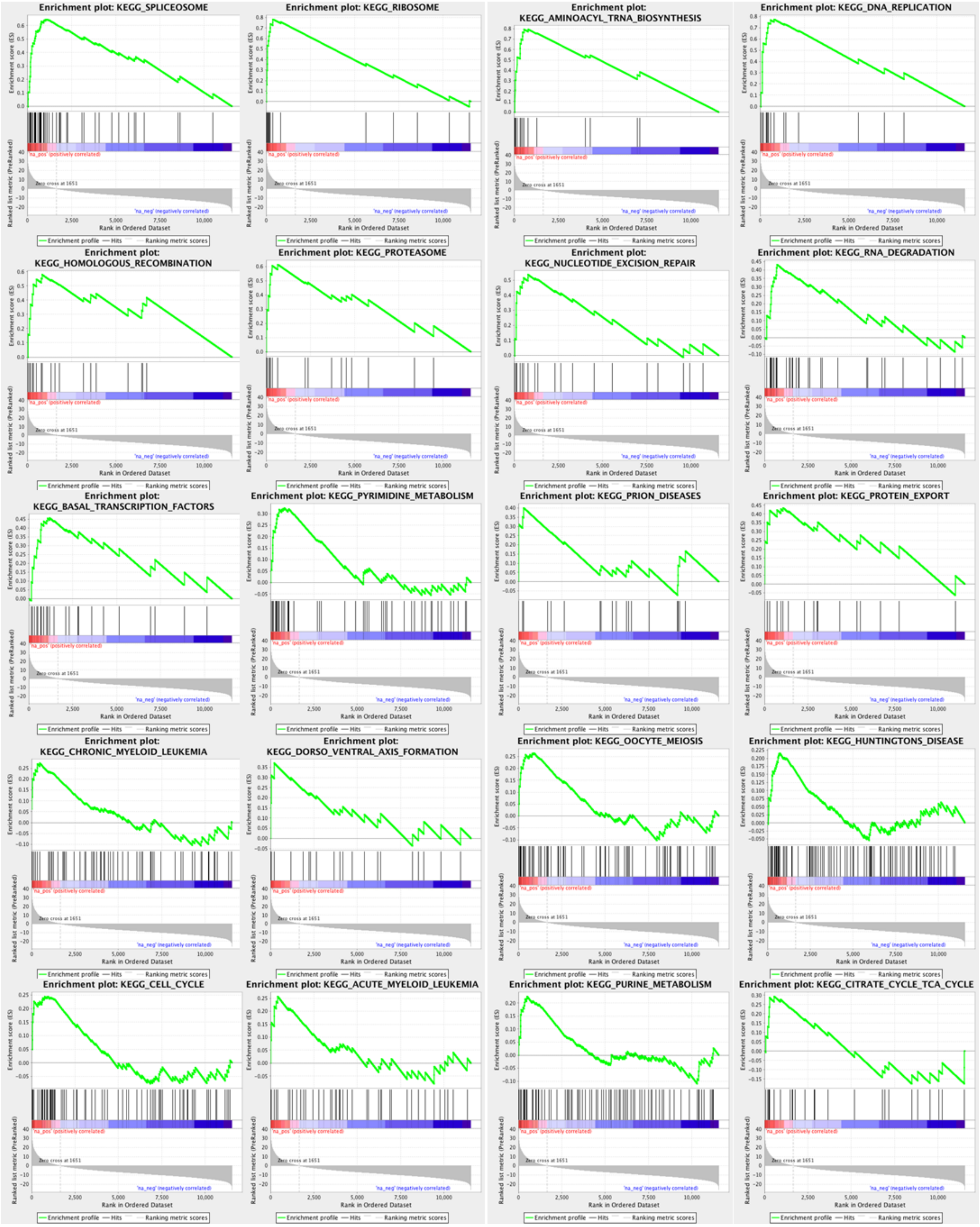

Supplementary Figure 6 Top 20 depleted pathways with Wang library

The gene level Bayesian factors (BF) generated from Wang library screen were used in the KEGG gene set enrichment analysis (GSEA). The enrichment plots of top 20 depleted pathways were listed.

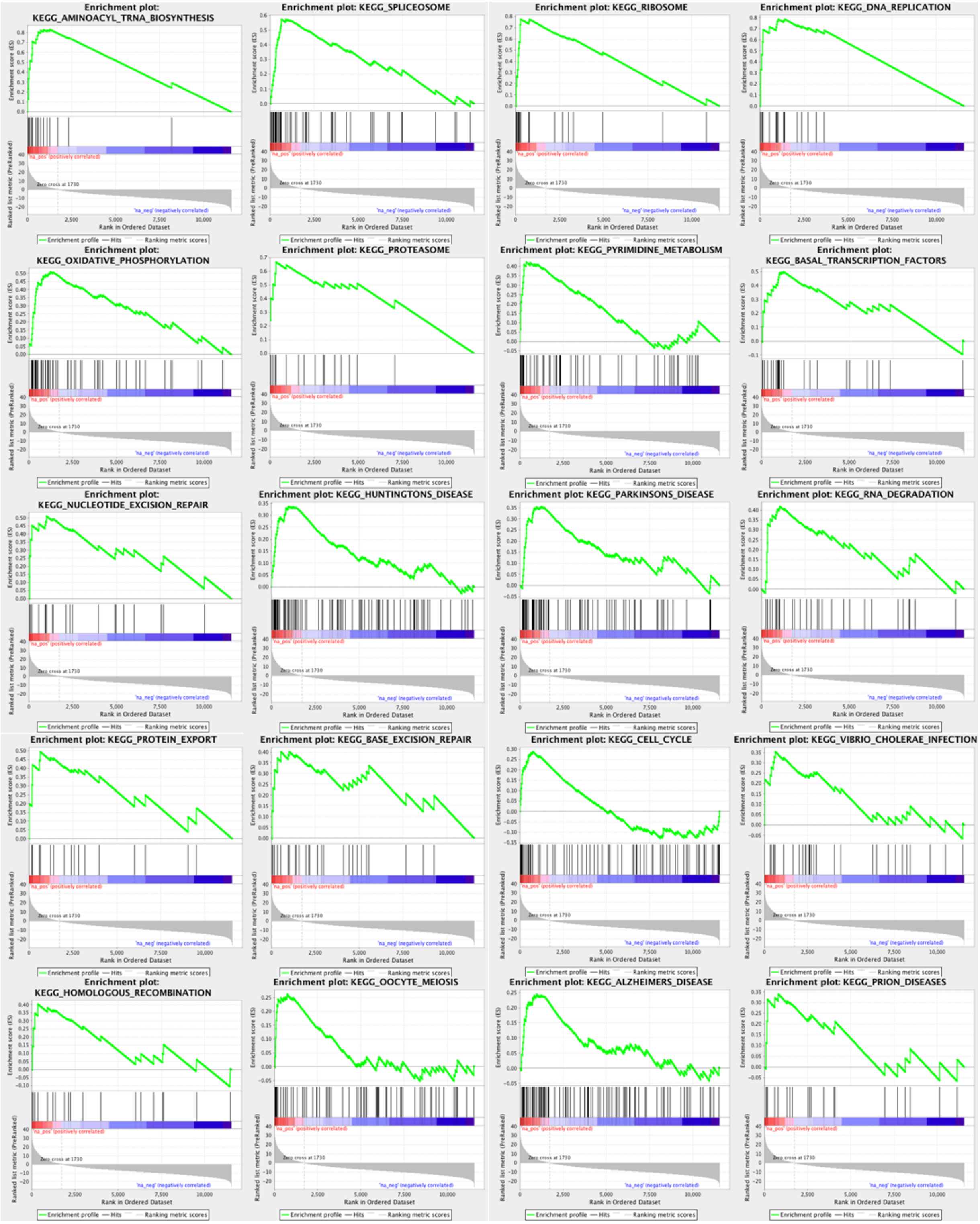

Supplementary Figure 7 Top 20 depleted pathways with GeCKO V2 library

The gene level Bayesian factors (BF) generated from GeCKOv2 library screen were used in the KEGG gene set enrichment analysis (GSEA). The enrichment plots of top 20 depleted pathways were listed.

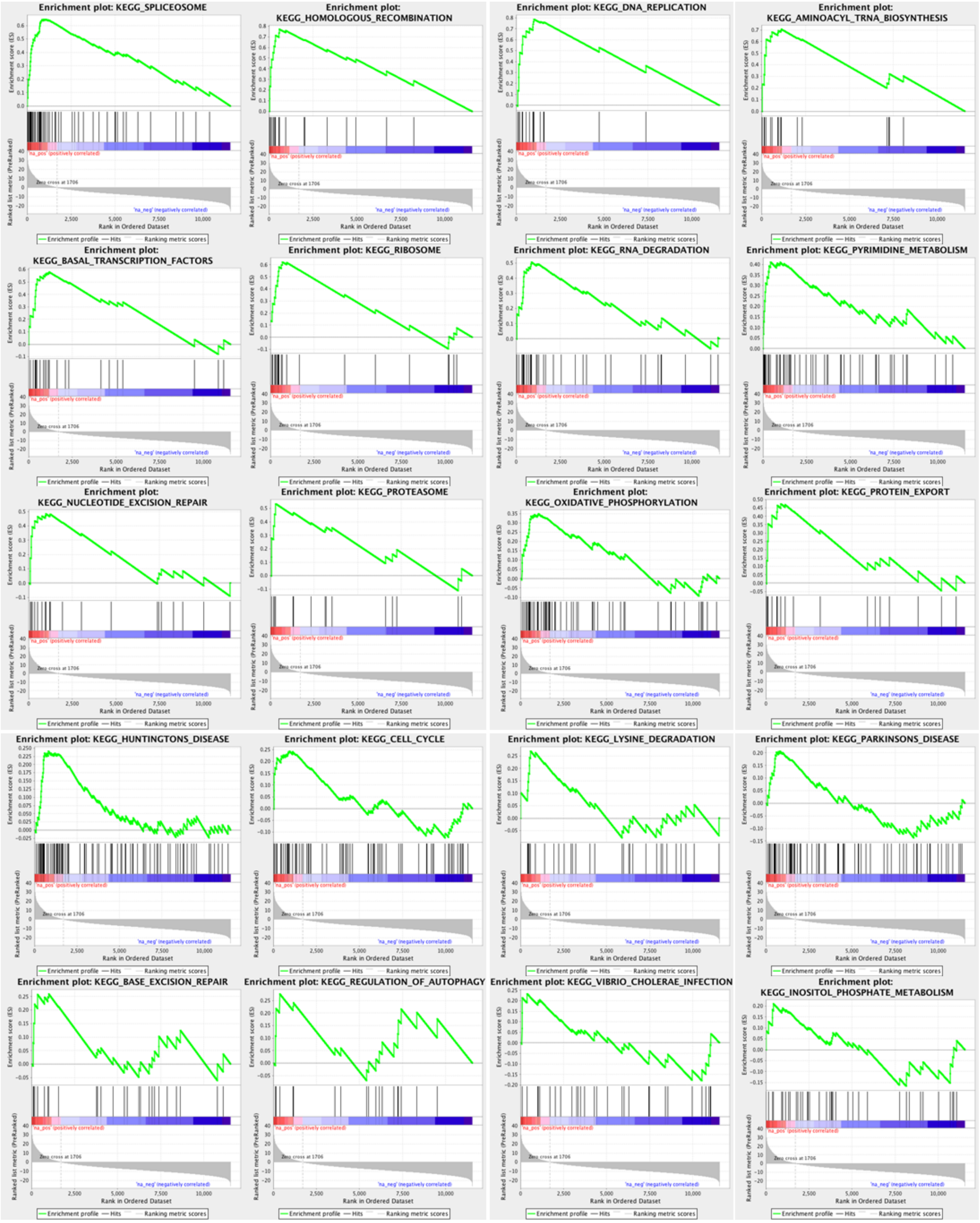

Supplementary Figure 8 Top 20 depleted pathways with Mini-human library

The gene level Bayesian factors (BF) generated from Mini-human library screen were used in the KEGG gene set enrichment analysis (GSEA). The enrichment plots of top 20 depleted pathways were listed.

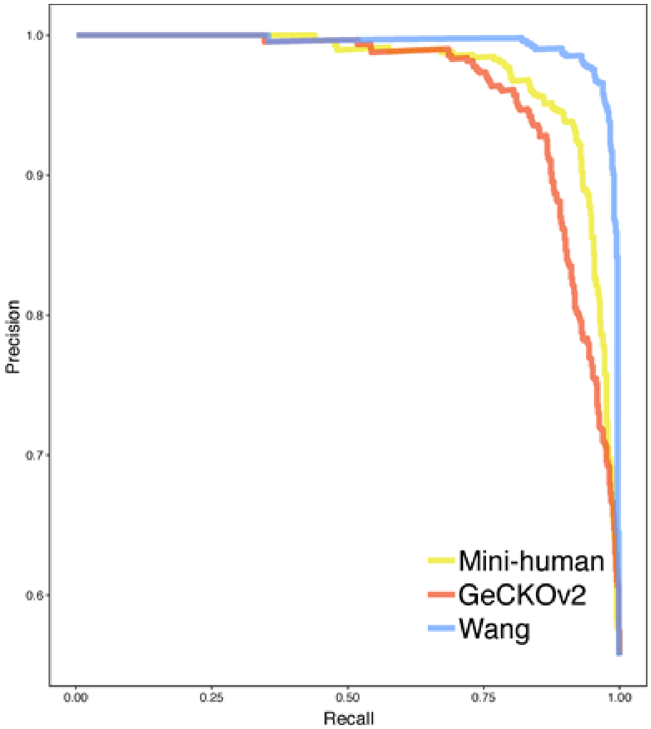

Supplementary Figure 9 The precision-recall curve plot of different CRISPR library screen

Blue *curve*: down-sampled Wang library (4 guides each gene). Red *curve*: GeCKO V2 library. Yellow *curve*: Mini-human library. Source data are provided as a Source Data file.

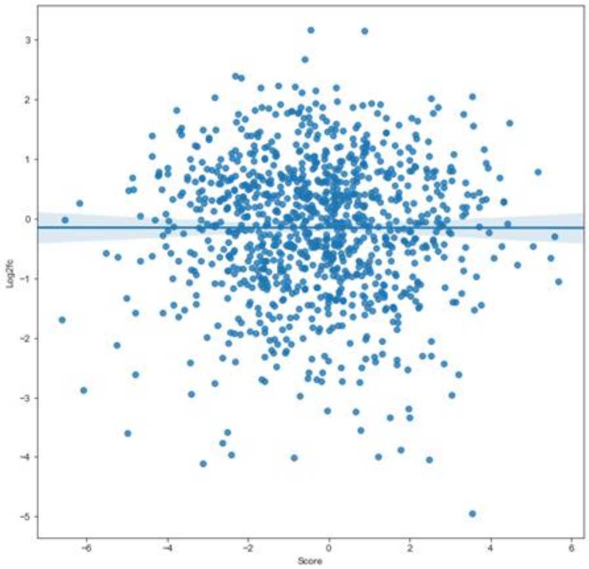

Supplementary Figure 10 Activity scores and depletion correlation of non-essential gene targeting guides.

Line of best fit is plotted and the shaded area is presented as SD*. (*Spearman's rho=-0.01, two-side p=0.65, Spearman's rank-order Correlation*).* Source data are provided as a Source Data file

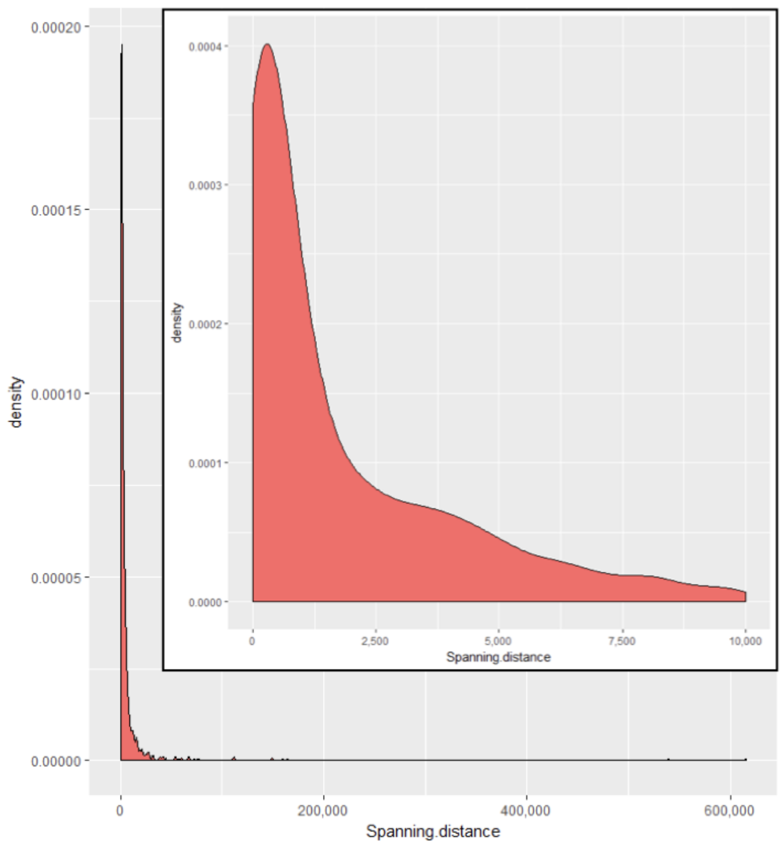

Supplementary Figure 11 Maximum distance of double strand breaks in AsCpf1 multiplexed library.

The maximum distance of double strand breaks was defined as the maximum distance of chromosomal coordinate between any two guides targeting the same gene. The upper enlarged right corner showed the distribution of the maximum distance shorter than 10 kilobases. Source data are provided as a Source Data file

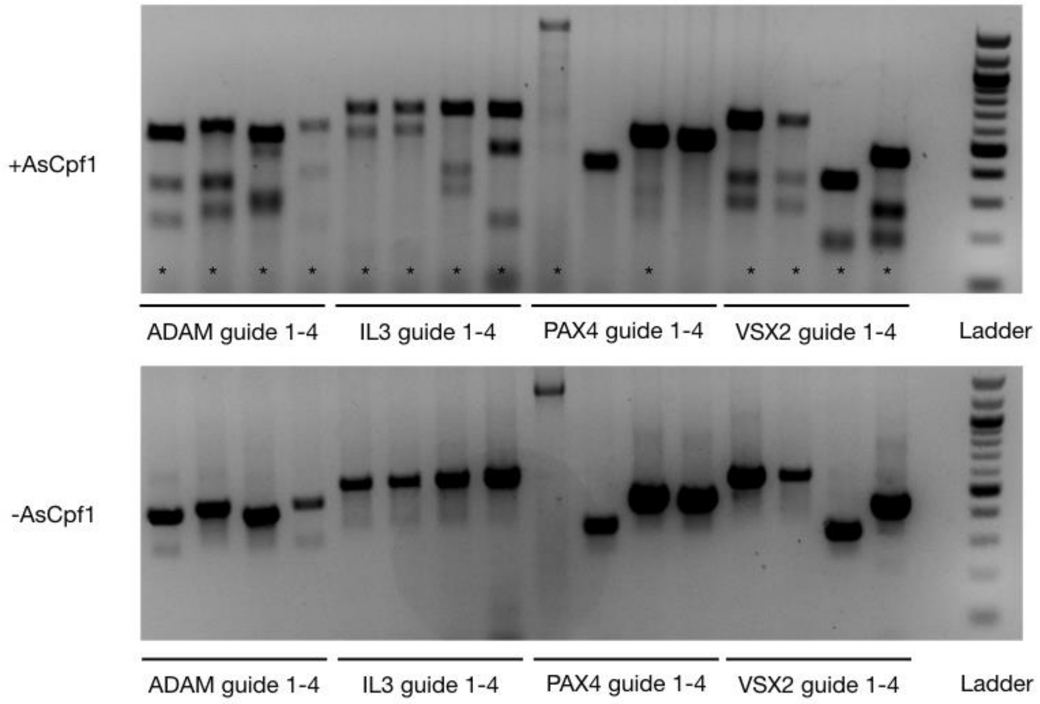

Supplementary Figure 12 T7E1 assay results of AsCpf1 on non-essential gene loci.

HEK293-T cells were transfected with AsCpf1-3xMYC and guides in complexed with lipofectamine 3000. Active guides are indicated by “*”. Upper panel: cells transfected with AsCpf1-3xMYC and guide. Lower panel: cells transfected with guides only. T7E1 assay products targeting loci (from left to right): ADAM, IL3, PAX4, VSX2. For each locus, 4 guides were selected from Mini-human library. Ladder: NEB 100bp DNA ladder. Source data are provided as a Source Data file.

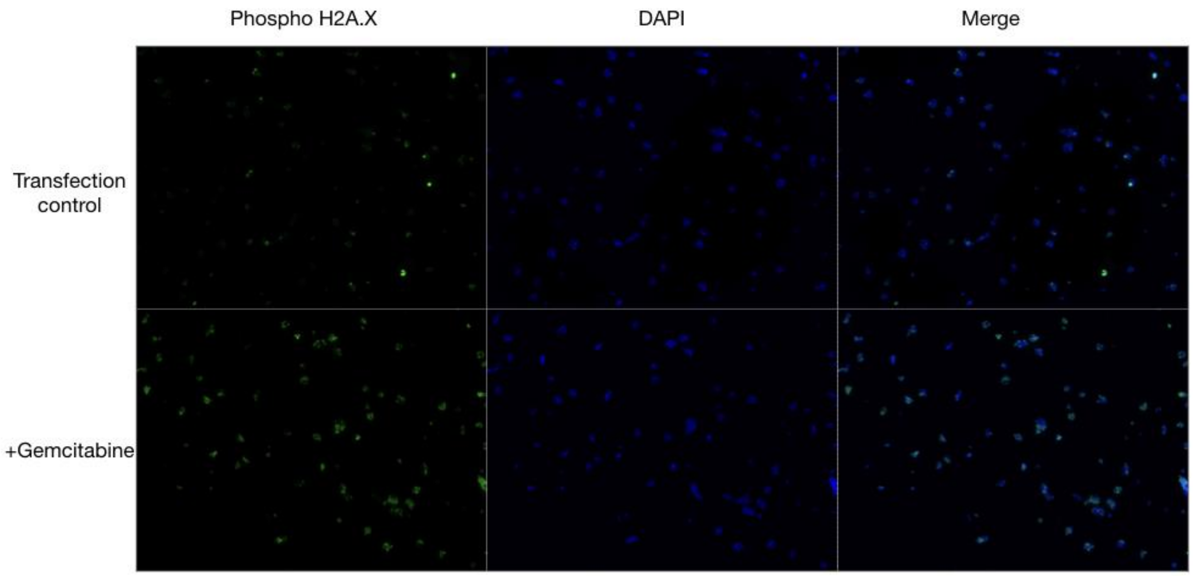

Supplementary Figure 13 Immunofluorescence microscopy validation of p-H2A.X antibody.

HEK293-T cells were transfected with AsCpf1-3xMYC and empty guide vector in complexed with Lipofectamine 3000 (Transfection control) or treated with 1-hour 100nM gemcitabine incubation (+Gemcitabine) and stained for H2A.X phosphorylation and nucleus. Source data are provided as a Source Data file.

	Restriction digestion (rearranged/total colony)	Gibson assembly (rearranged/total colony)
NEB Stable (Q5 polymerase)	0/8	2/10
DH10B (Q5 polymerase)	Not tested	8/11
NEB Stable (KOD polymerase)	0/9	3/9
DH10B (KOD polymerase)	0/10	Not tested

Supplementary.Table.1 Rate of plasmid rearrangement using different library cloning techniques

Oligo pools were PCR amplified with either KOD polymerase or NEB Q5 polymerase and then cloned into vector by either restrictive digestion or Gibson assembly. Ligation products were transformed into either

NEB stable strain or DH10B strain. Transformants were then subjected to sanger sequencing and mapped back to reference to detect whether vector recombination was present.

Sequence of LentiUniversal-Puro vector:

```
>LentiUniversal-Puro
CCCGGGTGCAAAGATGGATAAAGTTTTAAACAGAGAGGAATCTTTGCAGCTAATGGACCTTCTA
GGTCTTGAAAGGAGTGGGAATTGGCTCCGGTGCCCGTCAGTGGGCAGAGCGCACATCGCCCACA
GTCCCCGAGAAGTTGGGGGGAGGGGTCGGCAATTGATCCGGTGCCTAGAGAAGGTGGCGCGGGG
TAAACTGGGAAAGTGATGTCGTGTACTGGCTCCGCCTTTTTCCCGAGGGTGGGGGAGAACCGTA
TATAAGTGCAGTAGTCGCCGTGAACGTTCTTTTTCGCAACGGGTTTGCCGCCAGAACACAGGTA
AGTGCCGTGTGTGGTTCCCGCGGGCCTGGCCTCTTTACGGGTTATGGCCCTTGCGTGCCTTGAA
TTACTTCCACTGGCTGCAGTACGTGATTCTTGATCCCGAGCTTCGGGTTGGAAGTGGGTGGGAG
AGTTCGAGGCCTTGCGCTTAAGGAGCCCCTTCGCCTCGTGCTTGAGTTGAGGCCTGGCCTGGGC
GCTGGGGCCGCCGCGTGCGAATCTGGTGGCACCTTCGCGCCTGTCTCGCTGCTTTCGATAAGTC
TCTAGCCATTTAAAATTTTTGATGACCTGCTGCGACGCTTTTTTTCTGGCAAGATAGTCTTGTA
AATGCGGGCCAAGATCTGCACACTGGTATTTCGGTTTTTGGGGCCGCGGGCGGCGACGGGGCCC
GTGCGTCCCAGCGCACATGTTCGGCGAGGCGGGGCCTGCGAGCGCGGCCACCGAGAATCGGACG
GGGGTAGTCTCAAGCTGGCCGGCCTGCTCTGGTGCCTGGCCTCGCGCCGCCGTGTATCGCCCCG
CCCTGGGCGGCAAGGCTGGCCCGGTCGGCACCAGTTGCGTGAGCGGAAAGATGGCCGCTTCCCG
GCCCTGCTGCAGGGAGCTCAAAATGGAGGACGCGGCGCTCGGGAGAGCGGGCGGGTGAGTCACC
CACACAAAGGAAAAGGGCCTTTCCGTCCTCAGCCGTCGCTTCATGTGACTCCACGGAGTACCGG
GCGCCGTCCAGGCACCTCGATTAGTTCTCGAGCTTTTGGAGTACGTCGTCTTTAGGTTGGGGGG
AGGGGTTTTATGCGATGGAGTTTCCCCACACTGAGTGGGTGGAGACTGAAGTTAGGCCAGCTTG
GCACTTGATGTAATTCTCCTTGGAATTTGCCCTTTTTGAGTTTGGATCTTGGTTCATTCTCAAG
CCTCAGACAGTGGTTCAAAGTTTTTTTCTTCCATTTCAGGTGTCGTGACGTACGGCCACCATGA
CCGAGTACAAGCCCACGGTGCGCCTCGCCACCCGCGACGACGTCCCCAGGGCCGTACGCACCCT
CGCCGCCGCGTTCGCCGACTACCCCGCCACGCGCCACACCGTCGATCCGGACCGCCACATCGAG
CGGGTCACCGAGCTGCAAGAACTCTTCCTCACGCGCGTCGGGCTCGACATCGGCAAGGTGTGGG
TCGCGGACGACGGCGCCGCCGTGGCGGTCTGGACCACGCCGGAGAGCGTCGAAGCGGGGGCGGT
GTTCGCCGAGATCGGCCCGCGCATGGCCGAGTTGAGCGGTTCCCGGCTGGCCGCGCAGCAACAG
ATGGAAGGCCTCCTGGCGCCGCACCGGCCCAAGGAGCCCGCGTGGTTCCTGGCCACCGTCGGAG
TCTCGCCCGACCACCAGGGCAAGGGTCTGGGCAGCGCCGTCGTGCTCCCCGGAGTGGAGGCGGC
CGAGCGCGCCGGGGTGCCCGCCTTCCTGGAGACCTCCGCGCCCCGCAACCTCCCCTTCTACGAG
CGGCTCGGCTTCACCGTCACCGCCGACGTCGAGGTGCCCGAAGGACCGCGCACCTGGTGCATGA
CCCGCAAGCCCGGTGCCTGAACGCGTTAAGTCGACAATCAACCTCTGGATTACAAAATTTGTGA
AAGATTGACTGGTATTCTTAACTATGTTGCTCCTTTTACGCTATGTGGATACGCTGCTTTAATG
CCTTTGTATCATGCTATTGCTTCCCGTATGGCTTTCATTTTCTCCTCCTTGTATAAATCCTGGT
TGCTGTCTCTTTATGAGGAGTTGTGGCCCGTTGTCAGGCAACGTGGCGTGGTGTGCACTGTGTT
TGCTGACGCAACCCCCACTGGTTGGGGCATTGCCACCACCTGTCAGCTCCTTTCCGGGACTTTC
GCTTTCCCCCTCCCTATTGCCACGGCGGAACTCATCGCCGCCTGCCTTGCCCGCTGCTGGACAG
GGGCTCGGCTGTTGGGCACTGACAATTCCGTGGTGTTGTCGGGGAAATCATCGTCCTTTCCTTG
GCTGCTCGCCTGTGTTGCCACCTGGATTCTGCGCGGGACGTCCTTCTGCTACGTCCCTTCGGCC
CTCAATCCAGCGGACCTTCCTTCCCGCGGCCTGCTGCCGGCTCTGCGGCCTCTTCCGCGTCTTC
GCCTTCGCCCTCAGACGAGTCGGATCTCCCTTTGGGCCGCCTCCCCGCGTCGACTTTAAGACCA
ATGACTTACAAGGCAGCTGTAGATCTTAGCCACTTTTTAAAAGAAAAGGGGGGACTGGAAGGGC
TAATTCACTCCCAACGAAGACAAGATCTGCTTTTTGCTTGTACTGGGTCTCTCTGGTTAGACCA
```

GATCTGAGCCTGGGAGCTCTCTGGCTAACTAGGGAACCCACTGCTTAAGCCTCAATAAAGCTTG
CCTTGAGTGCTTCAAGTAGTGTGTGCCCGTCTGTTGTGTGACTCTGGTAACTAGAGATCCCTCA
GACCCTTTTAGTCAGTGTGGAAAATCTCTAGCAGTACGTATAGTAGTTCATGTCATCTTATTAT
TCAGTATTTATAACTTGCAAAGAAATGAATATCAGAGAGTGAGAGGAACTTGTTTATTGCAGCT
TATAATGGTTACAAATAAAGCAATAGCATCACAAATTTCACAAATAAAGCATTTTTTTCACTGC
ATTCTAGTTGTGGTTTGTCCAAACTCATCAATGTATCTTATCATGTCTGGCTCTAGCTATCCCG
CCCCTAACTCCGCCCATCCCGCCCCTAACTCCGCCCAGTTCCGCCCATTCTCCGCCCCATGGCT
GACTAATTTTTTTTATTTATGCAGAGGCCGAGGCCGCCTCGGCCTCTGAGCTATTCCAGAAGTA
GTGAGGAGGCTTTTTTGGAGGCCTAGGGACGTACCCAATTCGCCCTATAGTGAGTCGTATTACG
CGCGCTCACTGGCCGTCGTTTTACAACGTCGTGACTGGGAAAACCCTGGCGTTACCCAACTTAA
TCGCCTTGCAGCACATCCCCCTTTCGCCAGCTGGCGTAATAGCGAAGAGGCCCGCACCGATCGC
CCTTCCCAACAGTTGCGCAGCCTGAATGGCGAATGGGACGCGCCCTGTAGCGGCGCATTAAGCG
CGGCGGGTGTGGTGGTTACGCGCAGCGTGACCGCTACACTTGCCAGCGCCCTAGCGCCCGCTCC
TTTCGCTTTCTTCCCTTCCTTTCTCGCCACGTTCGCCGGCTTTCCCCGTCAAGCTCTAAATCGG
GGGCTCCCTTTAGGGTTCCGATTTAGTGCTTTACGGCACCTCGACCCCAAAAAACTTGATTAGG
GTGATGGTTCACGTAGTGGGCCATCGCCCTGATAGACGGTTTTTCGCCCTTTGACGTTGGAGTC
CACGTTCTTTAATAGTGGACTCTTGTTCCAAACTGGAACAACACTCAACCCTATCTCGGTCTAT
TCTTTTGATTTATAAGGGATTTTGCCGATTTCGGCCTATTGGTTAAAAAATGAGCTGATTTAAC
AAAAATTTAACGCGAATTTTAACAAAATATTAACGCTTACAATTTAGGTGGCACTTTTCGGGGA
AATGTGCGCGGAACCCCTATTTGTTTATTTTTCTAAATACATTCAAATATGTATCCGCTCATGA
GACAATAACCCTGATAAATGCTTCAATAATATTGAAAAAGGAAGAGTATGAGTATTCAACATTT
CCGTGTCGCCCTTATTCCCTTTTTTGCGGCATTTTGCCTTCCTGTTTTTGCTCACCCAGAAACG
CTGGTGAAAGTAAAAGATGCTGAAGATCAGTTGGGTGCACGAGTGGGTTACATCGAACTGGATC
TCAACAGCGGTAAGATCCTTGAGAGTTTTCGCCCCGAAGAACGTTTTCCAATGATGAGCACTTT
TAAAGTTCTGCTATGTGGCGCGGTATTATCCCGTATTGACGCCGGGCAAGAGCAACTCGGTCGC
CGCATACACTATTCTCAGAATGACTTGGTTGAGTACTCACCAGTCACAGAAAAGCATCTTACGG
ATGGCATGACAGTAAGAGAATTATGCAGTGCTGCCATAACCATGAGTGATAACACTGCGGCCAA
CTTACTTCTGACAACGATCGGAGGACCGAAGGAGCTAACCGCTTTTTTGCACAACATGGGGGAT
CATGTAACTCGCCTTGATCGTTGGGAACCGGAGCTGAATGAAGCCATACCAAACGACGAGCGTG
ACACCACGATGCCTGTAGCAATGGCAACAACGTTGCGCAAACTATTAACTGGCGAACTACTTAC
TCTAGCTTCCCGGCAACAATTAATAGACTGGATGGAGGCGGATAAAGTTGCAGGACCACTTCTG
CGCTCGGCCCTTCCGGCTGGCTGGTTTATTGCTGATAAATCTGGAGCCGGTGAGCGTGGGTCTC
GCGGTATCATTGCAGCACTGGGGCCAGATGGTAAGCCCTCCCGTATCGTAGTTATCTACACGAC
GGGGAGTCAGGCAACTATGGATGAACGAAATAGACAGATCGCTGAGATAGGTGCCTCACTGATT
AAGCATTGGTAACTGTCAGACCAAGTTTACTCATATATACTTTAGATTGATTTAAAACTTCATT
TTTAATTTAAAAGGATCTAGGTGAAGATCCTTTTTGATAATCTCATGACCAAAATCCCTTAACG
TGAGTTTTCGTTCCACTGAGCGTCAGACCCCGTAGAAAAGATCAAAGGATCTTCTTGAGATCCT
TTTTTTCTGCGCGTAATCTGCTGCTTGCAAACAAAAAAACCACCGCTACCAGCGGTGGTTTGTT
TGCCGGATCAAGAGCTACCAACTCTTTTTCCGAAGGTAACTGGCTTCAGCAGAGCGCAGATACC
AAATACTGTTCTTCTAGTGTAGCCGTAGTTAGGCCACCACTTCAAGAACTCTGTAGCACCGCCT
ACATACCTCGCTCTGCTAATCCTGTTACCAGTGGCTGCTGCCAGTGGCGATAAGTCGTGTCTTA
CCGGGTTGGACTCAAGACGATAGTTACCGGATAAGGCGCAGCGGTCGGGCTGAACGGGGGGTTC
GTGCACACAGCCCAGCTTGGAGCGAACGACCTACACCGAACTGAGATACCTACAGCGTGAGCTA
TGAGAAAGCGCCACGCTTCCCGAAGGGAGAAAGGCGGACAGGTATCCGGTAAGCGGCAGGGTCG
GAACAGGAGAGCGCACGAGGGAGCTTCCAGGGGGAAACGCCTGGTATCTTTATAGTCCTGTCGG
GTTTCGCCACCTCTGACTTGAGCGTCGATTTTTGTGATGCTCGTCAGGGGGGCGGAGCCTATGG
AAAAACGCCAGCAACGCGGCCTTTTTACGGTTCCTGGCCTTTTGCTGGCCTTTTGCTCACATGT

TCTTTCCTGCGTTATCCCCTGATTCTGTGGATAACCGTATTACCGCCTTTGAGTGAGCTGATAC
CGCTCGCCGCAGCCGAACGACCGAGCGCAGCGAGTCAGTGAGCGAGGAAGCGGAAGAGCGCCCA
ATACGCAAACCGCCTCTCCCCGCGCGTTGGCCGATTCATTAATGCAGCTGGCACGACAGGTTTC
CCGACTGGAAAGCGGGCAGTGAGCGCAACGCAATTAATGTGAGTTAGCTCACTCATTAGGCACC
CCAGGCTTTACACTTTATGCTTCCGGCTCGTATGTTGTGTGGAATTGTGAGCGGATAACAATTT
CACACAGGAAACAGCTATGACCATGATTACGCCAAGCGCGCAATTAACCCTCACTAAAGGGAAC
AAAAGCTGGAGCTGCAAGCTTAATGTAGTCTTATGCAATACTCTTGTAGTCTTGCAACATGGTA
ACGATGAGTTAGCAACATGCCTTACAAGGAGAGAAAAAGCACCGTGCATGCCGATTGGTGGAAG
TAAGGTGGTACGATCGTGCCTTATTAGGAAGGCAACAGACGGGTCTGACATGGATTGGACGAAC
CACTGAATTGCCGCATTGCAGAGATATTGTATTTAAGTGCCTAGCTCGATACATAAACGGGTCT
CTCTGGTTAGACCAGATCTGAGCCTGGGAGCTCTCTGGCTAACTAGGGAACCCACTGCTTAAGC
CTCAATAAAGCTTGCCTTGAGTGCTTCAAGTAGTGTGTGCCCGTCTGTTGTGTGACTCTGGTAA
CTAGAGATCCCTCAGACCCTTTTAGTCAGTGTGGAAAATCTCTAGCAGTGGCGCCCGAACAGGG
ACTTGAAAGCGAAAGGGAAACCAGAGGAGCTCTCTCGACGCAGGACTCGGCTTGCTGAAGCGCG
CACGGCAAGAGGCGAGGGGCGGCGACTGGTGAGTACGCCAAAAATTTTGACTAGCGGAGGCTAG
AAGGAGAGAGATGGGTGCGAGAGCGTCAGTATTAAGCGGGGGAGAATTAGATCGCGATGGGAAA
AAATTCGGTTAAGGCCAGGGGGAAAGAAAAAATATAAATTAAAACATATAGTATGGGCAAGCAG
GGAGCTAGAACGATTCGCAGTTAATCCTGGCCTGTTAGAAACATCAGAAGGCTGTAGACAAATA
CTGGGACAGCTACAACCATCCCTTCAGACAGGATCAGAAGAACTTAGATCATTATATAATACAG
TAGCAACCCTCTATTGTGTGCATCAAAGGATAGAGATAAAAGACACCAAGGAAGCTTTAGACAA
GATAGAGGAAGAGCAAAACAAAAGTAAGACCACCGCACAGCAAGCGGCCGCTGATCTTCAGACC
TGGAGGAGGAGATATGAGGGACAATTGGAGAAGTGAATTATATAAATATAAAGTAGTAAAAATT
GAACCATTAGGAGTAGCACCCACCAAGGCAAAGAGAAGAGTGGTGCAGAGAGAAAAAAGAGCAG
TGGGAATAGGAGCTTTGTTCCTTGGGTTCTTGGGAGCAGCAGGAAGCACTATGGGCGCAGCGTC
AATGACGCTGACGGTACAGGCCAGACAATTATTGTCTGGTATAGTGCAGCAGCAGAACAATTTG
CTGAGGGCTATTGAGGCGCAACAGCATCTGTTGCAACTCACAGTCTGGGGCATCAAGCAGCTCC
AGGCAAGAATCCTGGCTGTGGAAAGATACCTAAAGGATCAACAGCTCCTGGGGATTTGGGGTTG
CTCTGGAAAACTCATTTGCACCACTGCTGTGCCTTGGAATGCTAGTTGGAGTAATAAATCTCTG
GAACAGATTTGGAATCACACGACCTGGATGGAGTGGGACAGAGAAATTAACAATTACACAAGCT
TAATACACTCCTTAATTGAAGAATCGCAAAACCAGCAAGAAAAGAATGAACAAGAATTATTGGA
ATTAGATAAATGGGCAAGTTTGTGGAATTGGTTTAACATAACAAATTGGCTGTGGTATATAAAA
TTATTCATAATGATAGTAGGAGGCTTGGTAGGTTTAAGAATAGTTTTTGCTGTACTTTCTATAG
TGAATAGAGTTAGGCAGGGATATTCACCATTATCGTTTCAGACCCACCTCCCAACCCCGAGGGG
ACCCAGAGAGGGCCTATTTCCCATGATTCCTTCATATTTGCATATACGATACAAGGCTGTTAGA
GAGATAATTAGAATTAATTTGACTGTAAACACAAAGATATTAGTACAAAATACGTGACGTAGAA
AGTAATAATTTCTTGGGTAGTTTGCAGTTTTAAAATTATGTTTTAAAATGGACTATCATATGCT
TACCGTAACTTGAAAGTATTTCGATTTCTTGGCTTTATATATCTTGTGGGGAGACGAAAGGACG
AAACACCGGAGACGGTTGTAAATGAGCACACAAAATACACATGCTAAAATATTATATTCTATGA
CCTTTATAAAATCAACCAAAATCTTCTTTTTAATAACTTTAGTATCAATAATTAGAATTTTTAT
GTTCCTTTTTGCAAACTTTTAATAAAAATGAGCAAAATAAAAAAACGCTAGTTTTAGTAACTCG
CGTTGTTTTCTTCACCTTTAATAATAGCTACTCCACCACTTGTTCCTAAGCGGTCAGCTCCTGC
TTCAATCATTTTTTGAGCATCTTCAAATGTTCTAACTCCACCAGCTGCTTTAACTAAAGCATTG
TCTTTAACAACTGACTTCATTAGTTTAACATCTTCAAATGTTGCACCTGATTTTGAAAATCCTG
TTGATGTTTTAACAAATTCTAATCCAGCTTCAACAGCTATTTCACAAGCTTTCATGATTTCTTC
TTTTGTTAATAAACAATTTTCCATAATACATTTAACAACATGTGATCCAGCTGCTTTTTTTACA
GCTTTCATGTCTTCTAAAACTAATTCATAATTTTTGTCTTTTAATGCACCAATATTTAATACCA
TATCAATTTCTGTTGCACCATCTTTAATTGCTTCAGAAACTTCGAATGCTTTTGTAGCTGTTGT

GCATGCACCTAGAGGAAAACCTACAACATTTGTTATTCCTACATTTGTGCCTTTTAATAATTCT
TTACAATAGCTTGTTCAATATGAATTAACACAAACTGTTGCAAAATCAAATTCAATTGCTTCAT
CACATAATTGTTTAATTTCAGCTTTCGTAGCATCTTGTTTTAATAATGTGTGATCTATATATTT
GTTTAGTTTCATTTTTTCTCCTATATATTCATTTTTAATTTTAATTCTTTAATAATTTCGTCTA
CTTTAACTTTAGCGTTTTGAACAGATTCACCAACACCTATAAAATAAATTTTTAGTTTAGGTTC
AGTTCCACTTGGGCGAACAGCAAATCATGACTTATCTTCTAAATAAAATTTTAGTAAGTCTTGT
CCTGGCATATTATACATTCCATCGATGTAGTCTTCAACATTAACAACTTTAAGTCCAGCAATTT
GAGTTAAGGGTGTTGCTCTCAATGATTTCATTAATGGTTCAATTTTTAATTTCTTTTCTTCTGG
TTTAAAATTCAAGTTTAAAGTGAAAGTGTAATATGCACCCATTTCTTTAAATAAATCTTCTAAA
TAGTCTACTAATGTTTTATTTTGTTTTTTATAAAATCAAGCAGCCTCTGCTATTAATATAGAAG
CTTGTATTCCATCTTTATCTCTAGCTGAGTCATCAATTACATATCCATAACTTTCTTCATAAGC
AAAAACAAAATTTAATCCGTTATCTTCTTCTTTAGCAATTTCTCTACCCATTCATTTAAATCCA
GTTAAAGTTTTTACAATATTAACTCCATATTTTTCATGAGCGATTCTATCACCCAAATCACTTG
TTACAAAACTTGAATATAGAGCCGGATTTTTTGGAATGCTATTTAAGCGTTTTAGATTTGATAA
TTTTCAATCAATTAAAATTGGTCCTGTTTGATTTCCATCTAATCTTACAAAATGACCATCATGT
TTTATTGCCATTCCAAATCTGTCAGCATCTGGGTCATTCATAATAATAATATCTGCATCATGTT
TAATACCATATTCAAGCGGTATTTTTCATGCAGGATCAAATTCTGGATTTGGATTTACAACATT
TTTAAATGTTTCATCTTCAAATGCATGCTCTTCAACCTCAATAACGTTATATCCTGATTCACGT
AATATTTTTGGGGTAAATTTAGTTCCTGTTCCATTAACTGCGCTAAAAATAATTTTTAAATCTT
TTTTAGCTTCTTGCTCTTTTTTGTACGTCTCTGTTTTAGAGCTAGAAATAGCAAGTTAAAATAA
GGCTAGTCCGTTATCAACTTGAAAAAGTGGCACCGAGTCGGTGCTTTTTTAATTAAGGATCCCC
TCGGCGTCTCTGGTTAAGCTTGGCGTAACTAGATCTTGAGACAAATGGCAGTATTCATCCACAA
TTTTAAAAGAAAAGGGGGGATTGGGGGGTACAGTGCAGGGGAAAGAATAGTAGACATAATAGCA
ACAGACATACAAACTAAAGAATTACAAAAACAAATTACAAAAATTCAAAATTTTCGGGTTTATT
ACAGGGACAGCAGAGATCCACTTTGGCGCCGGCTCGAGGGGG

Sequence of AsCpf1-3xMYC vector:

>LentiAsCpf1-3xMYC-blast
GTCGACGGATCGGGAGATCTCCCGATCCCCTATGGTGCACTCTCAGTACAATCTGCTCTGATGC
CGCATAGTTAAGCCAGTATCTGCTCCCTGCTTGTGTGTTGGAGGTCGCTGAGTAGTGCGCGAGC
AAAATTTAAGCTACAACAAGGCAAGGCTTGACCGACAATTGCATGAAGAATCTGCTTAGGGTTA
GGCGTTTTGCGCTGCTTCGCGATGTACGGGCCAGATATACGCGTTGACATTGATTATTGACTAG
TTATTAATAGTAATCAATTACGGGGTCATTAGTTCATAGCCCATATATGGAGTTCCGCGTTACA
TAACTTACGGTAAATGGCCCGCCTGGCTGACCGCCCAACGACCCCCGCCCATTGACGTCAATAA
TGACGTATGTTCCCATAGTAACGCCAATAGGGACTTTCCATTGACGTCAATGGGTGGAGTATTT
ACGGTAAACTGCCCACTTGGCAGTACATCAAGTGTATCATATGCCAAGTACGCCCCCTATTGAC
GTCAATGACGGTAAATGGCCCGCCTGGCATTATGCCCAGTACATGACCTTATGGGACTTTCCTA
CTTGGCAGTACATCTACGTATTAGTCATCGCTATTACCATGGTGATGCGGTTTTGGCAGTACAT
CAATGGGCGTGGATAGCGGTTTGACTCACGGGGATTTCCAAGTCTCCACCCCATTGACGTCAAT
GGGAGTTTGTTTTGGCACCAAAATCAACGGGACTTTCCAAAATGTCGTAACAACTCCGCCCCAT
TGACGCAAATGGGCGGTAGGCGTGTACGGTGGGAGGTCTATATAAGCAGCGCGTTTTGCCTGTA
CTGGGTCTCTCTGGTTAGACCAGATCTGAGCCTGGGAGCTCTCTGGCTAACTAGGGAACCCACT
GCTTAAGCCTCAATAAAGCTTGCCTTGAGTGCTTCAAGTAGTGTGTGCCCGTCTGTTGTGTGAC
TCTGGTAACTAGAGATCCCTCAGACCCTTTTAGTCAGTGTGGAAAATCTCTAGCAGTGGCGCCC
GAACAGGGACTTGAAAGCGAAAGGGAAACCAGAGGAGCTCTCTCGACGCAGGACTCGGCTTGCT
GAAGCGCGCACGGCAAGAGGCGAGGGGCGGCGACTGGTGAGTACGCCAAAAATTTTGACTAGCG

GAGGCTAGAAGGAGAGAGATGGGTGCGAGAGCGTCAGTATTAAGCGGGGGAGAATTAGATCGCG
ATGGGAAAAAATTCGGTTAAGGCCAGGGGGAAAGAAAAAATATAAATTAAAACATATAGTATGG
GCAAGCAGGGAGCTAGAACGATTCGCAGTTAATCCTGGCCTGTTAGAAACATCAGAAGGCTGTA
GACAAATACTGGGACAGCTACAACCATCCCTTCAGACAGGATCAGAAGAACTTAGATCATTATA
TAATACAGTAGCAACCCTCTATTGTGTGCATCAAAGGATAGAGATAAAAGACACCAAGGAAGCT
TTAGACAAGATAGAGGAAGAGCAAAACAAAAGTAAGACCACCGCACAGCAAGCGGCCGCTGATC
TTCAGACCTGGAGGAGGAGATATGAGGGACAATTGGAGAAGTGAATTATATAAATATAAAGTAG
TAAAAATTGAACCATTAGGAGTAGCACCCACCAAGGCAAAGAGAAGAGTGGTGCAGAGAGAAAA
AAGAGCAGTGGGAATAGGAGCTTTGTTCCTTGGGTTCTTGGGAGCAGCAGGAAGCACTATGGGC
GCAGCGTCAATGACGCTGACGGTACAGGCCAGACAATTATTGTCTGGTATAGTGCAGCAGCAGA
ACAATTTGCTGAGGGCTATTGAGGCGCAACAGCATCTGTTGCAACTCACAGTCTGGGGCATCAA
GCAGCTCCAGGCAAGAATCCTGGCTGTGGAAAGATACCTAAAGGATCAACAGCTCCTGGGGATT
TGGGGTTGCTCTGGAAAACTCATTTGCACCACTGCTGTGCCTTGGAATGCTAGTTGGAGTAATA
AATCTCTGGAACAGATTTGGAATCACACGACCTGGATGGAGTGGGACAGAGAAATTAACAATTA
CACAAGCTTAATACACTCCTTAATTGAAGAATCGCAAAACCAGCAAGAAAAGAATGAACAAGAA
TTATTGGAATTAGATAAATGGGCAAGTTTGTGGAATTGGTTTAACATAACAAATTGGCTGTGGT
ATATAAAATTATTCATAATGATAGTAGGAGGCTTGGTAGGTTTAAGAATAGTTTTTGCTGTACT
TTCTATAGTGAATAGAGTTAGGCAGGGATATTCACCATTATCGTTTCAGACCCACCTCCCAACC
CCGAGGGGACCCGACAGGCCCGAAGGAATAGAAGAAGAAGGTGGAGAGAGAGACAGAGACAGAT
CCATTCGATTAGTGAACGGATCGGCACTGCGTGCGCCAATTCTGCAGACAAATGGCAGTATTCA
TCCACAATTTTAAAAGAAAAGGGGGGATTGGGGGGTACAGTGCAGGGGAAAGAATAGTAGACAT
AATAGCAACAGACATACAAACTAAAGAATTACAAAAACAAATTACAAAAATTCAAAATTTTCGG
GTTTATTACAGGGACAGCAGAGATCCAGTTTGGTTAATTAGCTAGCTAGGTCTTGAAAGGAGTG
GGAATTGGCTCCGGTGCCCGTCAGTGGGCAGAGCGCACATCGCCCACAGTCCCCGAGAAGTTGG
GGGGAGGGGTCGGCAATTGATCCGGTGCCTAGAGAAGGTGGCGCGGGGTAAACTGGGAAAGTGA
TGTCGTGTACTGGCTCCGCCTTTTTCCCGAGGGTGGGGGAGAACCGTATATAAGTGCAGTAGTC
GCCGTGAACGTTCTTTTTCGCAACGGGTTTGCCGCCAGAACACAGGACCGGTGCCACCATGGCG
CCAGCGGCAAAGCGCGTAAAATTGGACCCAGCAGCCAAACGAGTAAAACTCGATCCGGCTGCTA
AACGAGTTAAGCTGGACACACAGTTCGAGGGCTTTACCAACCTGTATCAGGTGAGCAAGACACT
GCGGTTTGAGCTGATCCCACAGGGCAAGACCCTGAAGCACATCCAGGAGCAGGGCTTCATCGAG
GAGGACAAGGCCCGCAATGATCACTACAAGGAGCTGAAGCCCATCATCGATCGGATCTACAAGA
CCTATGCCGACCAGTGCCTGCAGCTGGTGCAGCTGGATTGGGAGAACCTGAGCGCCGCCATCGA
CTCCTATAGAAAGGAGAAAACCGAGGAGACAAGGAACGCCCTGATCGAGGAGCAGGCCACATAT
CGCAATGCCATCCACGACTACTTCATCGGCCGGACAGACAACCTGACCGATGCCATCAATAAGA
GACACGCCGAGATCTACAAGGGCCTGTTCAAGGCCGAGCTGTTTAATGGCAAGGTGCTGAAGCA
GCTGGGCACCGTGACCACAACCGAGCACGAGAACGCCCTGCTGCGGAGCTTCGACAAGTTTACA
ACCTACTTCTCCGGCTTTTATGAGAACAGGAAGAACGTGTTCAGCGCCGAGGATATCAGCACAG
CCATCCCACACCGCATCGTGCAGGACAACTTCCCCAAGTTTAAGGAGAATTGTCACATCTTCAC
ACGCCTGATCACCGCCGTGCCCAGCCTGCGGGAGCACTTTGAGAACGTGAAGAAGGCCATCGGC
ATCTTCGTGAGCACCTCCATCGAGGAGGTGTTTTCCTTCCCTTTTTATAACCAGCTGCTGACAC
AGACCCAGATCGACCTGTATAACCAGCTGCTGGGAGGAATCTCTCGGGAGGCAGGCACCGAGAA
GATCAAGGGCCTGAACGAGGTGCTGAATCTGGCCATCCAGAAGAATGATGAGACAGCCCACATC
ATCGCCTCCCTGCCACACAGATTCATCCCCCTGTTTAAGCAGATCCTGTCCGATAGGAACACCC
TGTCTTTCATCCTGGAGGAGTTTAAGAGCGACGAGGAAGTGATCCAGTCCTTCTGCAAGTACAA
GACACTGCTGAGAAACGAGAACGTGCTGGAGACAGCCGAGGCCCTGTTTAACGAGCTGAACAGC
ATCGACCTGACACACATCTTCATCAGCCACAAGAAGCTGGAGACAATCAGCAGCGCCCTGTGCG
ACCACTGGGATACACTGAGGAATGCCCTGTATGAGCGGAGAATCTCCGAGCTGACAGGCAAGAT

CACCAAGTCTGCCAAGGAGAAGGTGCAGCGCAGCCTGAAGCACGAGGATATCAACCTGCAGGAG
ATCATCTCTGCCGCAGGCAAGGAGCTGAGCGAGGCCTTCAAGCAGAAAACCAGCGAGATCCTGT
CCCACGCACACGCCGCCCTGGATCAGCCACTGCCTACAACCCTGAAGAAGCAGGAGGAGAAGGA
GATCCTGAAGTCTCAGCTGGACAGCCTGCTGGGCCTGTACCACCTGCTGGACTGGTTTGCCGTG
GATGAGTCCAACGAGGTGGACCCCGAGTTCTCTGCCCGGCTGACCGGCATCAAGCTGGAGATGG
AGCCTTCTCTGAGCTTCTACAACAAGGCCAGAAATTATGCCACCAAGAAGCCCTACTCCGTGGA
GAAGTTCAAGCTGAACTTTCAGATGCCTACACTGGCCTCTGGCTGGGACGTGAATAAGGAGAAG
AACAATGGCGCCATCCTGTTTGTGAAGAACGGCCTGTACTATCTGGGCATCATGCCAAAGCAGA
AGGGCAGGTATAAGGCCCTGAGCTTCGAGCCCACAGAGAAAACCAGCGAGGGCTTTGATAAGAT
GTACTATGACTACTTCCCTGATGCCGCCAAGATGATCCCAAAGTGCAGCACCCAGCTGAAGGCC
GTGACAGCCCACTTTCAGACCCACACAACCCCCATCCTGCTGTCCAACAATTTCATCGAGCCTC
TGGAGATCACAAAGGAGATCTACGACCTGAACAATCCTGAGAAGGAGCCAAAGAAGTTTCAGAC
AGCCTACGCCAAGAAAACCGGCGACCAGAAGGGCTACAGAGAGGCCCTGTGCAAGTGGATCGAC
TTCACAAGGGATTTTCTGTCCAAGTATACCAAGACAACCTCTATCGATCTGTCTAGCCTGCGGC
CATCCTCTCAGTATAAGGACCTGGGCGAGTACTATGCCGAGCTGAATCCCCTGCTGTACCACAT
CAGCTTCCAGAGAATCGCCGAGAAGGAGATCATGGATGCCGTGGAGACAGGCAAGCTGTACCTG
TTCCAGATCTATAACAAGGACTTTGCCAAGGGCCACCACGGCAAGCCTAATCTGCACACACTGT
ATTGGACCGGCCTGTTTTCTCCAGAGAACCTGGCCAAGACAAGCATCAAGCTGAATGGCCAGGC
CGAGCTGTTCTACCGCCCTAAGTCCAGGATGAAGAGGATGGCACACCGGCTGGGAGAGAAGATG
CTGAACAAGAAGCTGAAGGATCAGAAAACCCCAATCCCCGACACCCTGTACCAGGAGCTGTACG
ACTATGTGAATCACAGACTGTCCCACGACCTGTCTGATGAGGCCAGGGCCCTGCTGCCCAACGT
GATCACCAAGGAGGTGTCTCACGAGATCATCAAGGATAGGCGCTTTACCAGCGACAAGTTCTTT
TTCCACGTGCCTATCACACTGAACTATCAGGCCGCCAATTCCCCATCTAAGTTCAACCAGAGGG
TGAATGCCTACCTGAAGGAGCACCCCGAGACACCTATCATCGGCATCGATCGGGGCGAGAGAAA
CCTGATCTATATCACAGTGATCGACTCCACCGGCAAGATCCTGGAGCAGCGGAGCCTGAACACC
ATCCAGCAGTTTGATTACCAGAAGAAGCTGGACAACAGGGAGAAGGAGAGGGTGGCAGCAAGGC
AGGCCTGGTCTGTGGTGGGCACAATCAAGGATCTGAAGCAGGGCTATCTGAGCCAGGTCATCCA
CGAGATCGTGGACCTGATGATCCACTACCAGGCCGTGGTGGTGCTGGAGAACCTGAATTTCGGC
TTTAAGAGCAAGAGGACCGGCATCGCCGAGAAGGCCGTGTACCAGCAGTTCGAGAAGATGCTGA
TCGATAAGCTGAATTGCCTGGTGCTGAAGGACTATCCAGCAGAGAAAGTGGGAGGCGTGCTGAA
CCCATACCAGCTGACAGACCAGTTCACCTCCTTTGCCAAGATGGGCACCCAGTCTGGCTTCCTG
TTTTACGTGCCTGCCCCATATACATCTAAGATCGATCCCCTGACCGGCTTCGTGGACCCCTTCG
TGTGGAAAACCATCAAGAATCACGAGAGCCGCAAGCACTTCCTGGAGGGCTTCGACTTTCTGCA
CTACGACGTGAAAACCGGCGACTTCATCCTGCACTTTAAGATGAACAGAAATCTGTCCTTCCAG
AGGGGCCTGCCCGGCTTTATGCCTGCATGGGATATCGTGTTCGAGAAGAACGAGACACAGTTTG
ACGCCAAGGGCACCCCTTTCATCGCCGGCAAGAGAATCGTGCCAGTGATCGAGAATCACAGATT
CACCGGCAGATACCGGGACCTGTATCCTGCCAACGAGCTGATCGCCCTGCTGGAGGAGAAGGGC
ATCGTGTTCAGGGATGGCTCCAACATCCTGCCAAAGCTGCTGGAGAATGACGATTCTCACGCCA
TCGACACCATGGTGGCCCTGATCCGCAGCGTGCTGCAGATGCGGAACTCCAATGCCGCCACAGG
CGAGGACTATATCAACAGCCCCGTGCGCGATCTGAATGGCGTGTGCTTCGACTCCCGGTTTCAG
AACCCAGAGTGGCCCATGGACGCCGATGCCAATGGCGCCTACCACATCGCCCTGAAGGGCCAGC
TGCTGCTGAATCACCTGAAGGAGAGCAAGGATCTGAAGCTGCAGAACGGCATCTCCAATCAGGA
CTGGCTGGCCTACATCCAGGAGCTGCGCAACGGATCCGGCGCAACAAACTTCTCTCTGCTGAAA
CAAGCCGGAGATGTCGAAGAGAATCCTGGACCGATGGCCAAGCCTTTGTCTCAAGAAGAATCCA
CCCTCATTGAAAGAGCAACGGCTACAATCAACAGCATCCCCATCTCTGAAGACTACAGCGTCGC
CAGCGCAGCTCTCTCTAGCGACGGCCGCATCTTCACTGGTGTCAATGTATATCATTTTACTGGG
GGACCTTGTGCAGAACTCGTGGTGCTGGGCACTGCTGCTGCTGCGGCAGCTGGCAACCTGACTT

GTATCGTCGCGATCGGAAATGAGAACAGGGGCATCTTGAGCCCCTGCGGACGGTGCCGACAGGT
GCTTCTCGATCTGCATCCTGGGATCAAAGCCATAGTGAAGGACAGTGATGGACAGCCGACGGCA
GTTGGGATTCGTGAATTGCTGCCCTCTGGTTATGTGTGGGAGGGCTAAGAATTCGATATCAAGC
TTATCGGTAATCAACCTCTGGATTACAAAATTTGTGAAAGATTGACTGGTATTCTTAACTATGT
TGCTCCTTTTACGCTATGTGGATACGCTGCTTTAATGCCTTTGTATCATGCTATTGCTTCCCGT
ATGGCTTTCATTTTCTCCTCCTTGTATAAATCCTGGTTGCTGTCTCTTTATGAGGAGTTGTGGC
CCGTTGTCAGGCAACGTGGCGTGGTGTGCACTGTGTTTGCTGACGCAACCCCCACTGGTTGGGG
CATTGCCACCACCTGTCAGCTCCTTTCCGGGACTTTCGCTTTCCCCCTCCCTATTGCCACGGCG
GAACTCATCGCCGCCTGCCTTGCCCGCTGCTGGACAGGGGCTCGGCTGTTGGGCACTGACAATT
CCGTGGTGTTGTCGGGGAAATCATCGTCCTTTCCTTGGCTGCTCGCCTGTGTTGCCACCTGGAT
TCTGCGCGGGACGTCCTTCTGCTACGTCCCTTCGGCCCTCAATCCAGCGGACCTTCCTTCCCGC
GGCCTGCTGCCGGCTCTGCGGCCTCTTCCGCGTCTTCGCCTTCGCCCTCAGACGAGTCGGATCT
CCCTTTGGGCCGCCTCCCCGCATCGATACCGTCGACCTCGAGACCTAGAAAAACATGGAGCAAT
CACAAGTAGCAATACAGCAGCTACCAATGCTGATTGTGCCTGGCTAGAAGCACAAGAGGAGGAG
GAGGTGGGTTTTCCAGTCACACCTCAGGTACCTTTAAGACCAATGACTTACAAGGCAGCTGTAG
ATCTTAGCCACTTTTTAAAAGAAAAGGGGGGACTGGAAGGGCTAATTCACTCCCAACGAAGACA
AGATATCCTTGATCTGTGGATCTACCACACACAAGGCTACTTCCCTGATTGGCAGAACTACACA
CCAGGGCCAGGGATCAGATATCCACTGACCTTTGGATGGTGCTACAAGCTAGTACCAGTTGAGC
AAGAGAAGGTAGAAGAAGCCAATGAAGGAGAGAACACCCGCTTGTTACACCCTGTGAGCCTGCA
TGGGATGGATGACCCGGAGAGAGAAGTATTAGAGTGGAGGTTTGACAGCCGCCTAGCATTTCAT
CACATGGCCCGAGAGCTGCATCCGGACTGTACTGGGTCTCTCTGGTTAGACCAGATCTGAGCCT
GGGAGCTCTCTGGCTAACTAGGGAACCCACTGCTTAAGCCTCAATAAAGCTTGCCTTGAGTGCT
TCAAGTAGTGTGTGCCCGTCTGTTGTGTGACTCTGGTAACTAGAGATCCCTCAGACCCTTTTAG
TCAGTGTGGAAAATCTCTAGCAGGGCCCGTTTAAACCCGCTGATCAGCCTCGACTGTGCCTTCT
AGTTGCCAGCCATCTGTTGTTTGCCCCTCCCCCGTGCCTTCCTTGACCCTGGAAGGTGCCACTC
CCACTGTCCTTTCCTAATAAAATGAGGAAATTGCATCGCATTGTCTGAGTAGGTGTCATTCTAT
TCTGGGGGGTGGGGTGGGGCAGGACAGCAAGGGGGAGGATTGGGAAGACAATAGCAGGCATGCT
GGGGATGCGGTGGGCTCTATGGCTTCTGAGGCGGAAAGAACCAGCTGGGGCTCTAGGGGGTATC
CCCACGCGCCCTGTAGCGGCGCATTAAGCGCGGCGGGTGTGGTGGTTACGCGCAGCGTGACCGC
TACACTTGCCAGCGCCCTAGCGCCCGCTCCTTTCGCTTTCTTCCCTTCCTTTCTCGCCACGTTC
GCCGGCTTTCCCCGTCAAGCTCTAAATCGGGGGCTCCCTTTAGGGTTCCGATTTAGTGCTTTAC
GGCACCTCGACCCCAAAAAACTTGATTAGGGTGATGGTTCACGTAGTGGGCCATCGCCCTGATA
GACGGTTTTTCGCCCTTTGACGTTGGAGTCCACGTTCTTTAATAGTGGACTCTTGTTCCAAACT
GGAACAACACTCAACCCTATCTCGGTCTATTCTTTTGATTTATAAGGGATTTTGCCGATTTCGG
CCTATTGGTTAAAAAATGAGCTGATTTAACAAAAATTTAACGCGAATTAATTCTGTGGAATGTG
TGTCAGTTAGGGTGTGGAAAGTCCCCAGGCTCCCCAGCAGGCAGAAGTATGCAAAGCATGCATC
TCAATTAGTCAGCAACCAGGTGTGGAAAGTCCCCAGGCTCCCCAGCAGGCAGAAGTATGCAAAG
CATGCATCTCAATTAGTCAGCAACCATAGTCCCGCCCCTAACTCCGCCCATCCCGCCCCTAACT
CCGCCCAGTTCCGCCCATTCTCCGCCCCATGGCTGACTAATTTTTTTTATTTATGCAGAGGCCG
AGGCCGCCTCTGCCTCTGAGCTATTCCAGAAGTAGTGAGGAGGCTTTTTTGGAGGCCTAGGCTT
TTGCAAAAAGCTCCCGGGAGCTTGTATATCCATTTTCGGATCTGATCAGCACGTGTTGACAATT
AATCATCGGCATAGTATATCGGCATAGTATAATACGACAAGGTGAGGAACTAAACCATGGCCAA
GTTGACCAGTGCCGTTCCGGTGCTCACCGCGCGCGACGTCGCCGGAGCGGTCGAGTTCTGGACC
GACCGGCTCGGGTTCTCCCGGGACTTCGTGGAGGACGACTTCGCCGGTGTGGTCCGGGACGACG
TGACCCTGTTCATCAGCGCGGTCCAGGACCAGGTGGTGCCGGACAACACCCTGGCCTGGGTGTG
GGTGCGCGGCCTGGACGAGCTGTACGCCGAGTGGTCGGAGGTCGTGTCCACGAACTTCCGGGAC
GCCTCCGGGCCGGCCATGACCGAGATCGGCGAGCAGCCGTGGGGGCGGGAGTTCGCCCTGCGCG

ACCCGGCCGGCAACTGCGTGCACTTCGTGGCCGAGGAGCAGGACTGACACGTGCTACGAGATTT
CGATTCCACCGCCGCCTTCTATGAAAGGTTGGGCTTCGGAATCGTTTTCCGGGACGCCGGCTGG
ATGATCCTCCAGCGCGGGGATCTCATGCTGGAGTTCTTCGCCCACCCCAACTTGTTTATTGCAG
CTTATAATGGTTACAAATAAAGCAATAGCATCACAAATTTCACAAATAAAGCATTTTTTTCACT
GCATTCTAGTTGTGGTTTGTCCAAACTCATCAATGTATCTTATCATGTCTGTATACCGTCGACC
TCTAGCTAGAGCTTGGCGTAATCATGGTCATAGCTGTTTCCTGTGTGAAATTGTTATCCGCTCA
CAATTCCACACAACATACGAGCCGGAAGCATAAAGTGTAAAGCCTGGGGTGCCTAATGAGTGAG
CTAACTCACATTAATTGCGTTGCGCTCACTGCCCGCTTTCCAGTCGGGAAACCTGTCGTGCCAG
CTGCATTAATGAATCGGCCAACGCGCGGGGAGAGGCGGTTTGCGTATTGGGCGCTCTTCCGCTT
CCTCGCTCACTGACTCGCTGCGCTCGGTCGTTCGGCTGCGGCGAGCGGTATCAGCTCACTCAAA
GGCGGTAATACGGTTATCCACAGAATCAGGGGATAACGCAGGAAAGAACATGTGAGCAAAAGGC
CAGCAAAAGGCCAGGAACCGTAAAAAGGCCGCGTTGCTGGCGTTTTTCCATAGGCTCCGCCCCC
CTGACGAGCATCACAAAAATCGACGCTCAAGTCAGAGGTGGCGAAACCCGACAGGACTATAAAG
ATACCAGGCGTTTCCCCCTGGAAGCTCCCTCGTGCGCTCTCCTGTTCCGACCCTGCCGCTTACC
GGATACCTGTCCGCCTTTCTCCCTTCGGGAAGCGTGGCGCTTTCTCATAGCTCACGCTGTAGGT
ATCTCAGTTCGGTGTAGGTCGTTCGCTCCAAGCTGGGCTGTGTGCACGAACCCCCCGTTCAGCC
CGACCGCTGCGCCTTATCCGGTAACTATCGTCTTGAGTCCAACCCGGTAAGACACGACTTATCG
CCACTGGCAGCAGCCACTGGTAACAGGATTAGCAGAGCGAGGTATGTAGGCGGTGCTACAGAGT
TCTTGAAGTGGTGGCCTAACTACGGCTACACTAGAAGAACAGTATTTGGTATCTGCGCTCTGCT
GAAGCCAGTTACCTTCGGAAAAAGAGTTGGTAGCTCTTGATCCGGCAAACAAACCACCGCTGGT
AGCGGTGGTTTTTTTGTTTGCAAGCAGCAGATTACGCGCAGAAAAAAAGGATCTCAAGAAGATC
CTTTGATCTTTTCTACGGGGTCTGACGCTCAGTGGAACGAAAACTCACGTTAAGGGATTTTGGT
CATGAGATTATCAAAAAGGATCTTCACCTAGATCCTTTTAAATTAAAAATGAAGTTTTAAATCA
ATCTAAAGTATATATGAGTAAACTTGGTCTGACAGTTACCAATGCTTAATCAGTGAGGCACCTA
TCTCAGCGATCTGTCTATTTCGTTCATCCATAGTTGCCTGACTCCCCGTCGTGTAGATAACTAC
GATACGGGAGGGCTTACCATCTGGCCCCAGTGCTGCAATGATACCGCGAGACCCACGCTCACCG
GCTCCAGATTTATCAGCAATAAACCAGCCAGCCGGAAGGGCCGAGCGCAGAAGTGGTCCTGCAA
CTTTATCCGCCTCCATCCAGTCTATTAATTGTTGCCGGGAAGCTAGAGTAAGTAGTTCGCCAGT
TAATAGTTTGCGCAACGTTGTTGCCATTGCTACAGGCATCGTGGTGTCACGCTCGTCGTTTGGT
ATGGCTTCATTCAGCTCCGGTTCCCAACGATCAAGGCGAGTTACATGATCCCCCATGTTGTGCA
AAAAAGCGGTTAGCTCCTTCGGTCCTCCGATCGTTGTCAGAAGTAAGTTGGCCGCAGTGTTATC
ACTCATGGTTATGGCAGCACTGCATAATTCTCTTACTGTCATGCCATCCGTAAGATGCTTTTCT
GTGACTGGTGAGTACTCAACCAAGTCATTCTGAGAATAGTGTATGCGGCGACCGAGTTGCTCTT
GCCCGGCGTCAATACGGGATAATACCGCGCCACATAGCAGAACTTTAAAAGTGCTCATCATTGG
AAAACGTTCTTCGGGGCGAAAACTCTCAAGGATCTTACCGCTGTTGAGATCCAGTTCGATGTAA
CCCACTCGTGCACCCAACTGATCTTCAGCATCTTTTACTTTCACCAGCGTTTCTGGGTGAGCAA
AAACAGGAAGGCAAAATGCCGCAAAAAAGGGAATAAGGGCGACACGGAAATGTTGAATACTCAT
ACTCTTCCTTTTTCAATATTATTGAAGCATTTATCAGGGTTATTGTCTCATGAGCGGATACATA
TTTGAATGTATTTAGAAAAATAAACAAATAGGGGTTCCGCGCACATTTCCCCGAAAAGTGCCAC
CTGAC

Highlight:AsCpf1-3xMYC sequence
