# Supplementary material for: Pooled library screening with multiplexed Cpf1 library
Source: Nat Commun. 2019 Jul 17;10:3144. doi: 10.1038/s41467-019-10963-x (PMC6637147; doi:10.1038/s41467-019-10963-x)
Supplement: Supplementary file 2 — Description of Additional Supplementary Files [file 41467_2019_10963_MOESM2_ESM.pdf]

Description of Additional Supplementary Files

Title: Supplementary Data 1

Description: Library information for “Mini-Human, 1st column is the gene symbol, 2nd column is its ensemble ID, 3rd column indicates how many guides are designed, for genes that has a guides number less than 4, a stuffer non-coding pseudo-guide is added. Column 4 is the oligo information for pooled oligo synthesis, column 5 to 8 contain guide information for each guide position.

Title: Supplementary Data 2

Description: Library information for AsCpf1 based monocistronic benchmark library.

Title: Supplementary Data 3

Description: Library information for SpCas9 based benchmark library

Title: Supplementary Data 4

Description: Library information for AsCpf1 based multiplexed benchmark library

Title: Supplementary Data 5

Description: Primers used for T7E1 assays
